# Mimicking lipolytic, adipogenic, and secretory capacities of human subcutaneous adipose tissue by spheroids from distinct subpopulations of adipose stromal/stem cells

**DOI:** 10.3389/fcell.2023.1219218

**Published:** 2023-09-29

**Authors:** Isis Côrtes, Gutemberg Alves, Cesar Claudio-Da-Silva, Leandra Santos Baptista

**Affiliations:** ^1^ Federal University of Rio de Janeiro, Campus UFRJ Duque de Caxias Professor Geraldo Cidade, Rio de Janeiro, Brazil; ^2^ Laboratory of Tissue Bioengineering, National Institute of Metrology, Quality and Technology (Inmetro), Rio de Janeiro, Brazil; ^3^ Post-graduation Program in Biotechnology, National Institute of Metrology, Quality and Technology (Inmetro), Rio de Janeiro, Brazil; ^4^ Cell and Molecular Biology Department, Institute of Biology, Fluminense Federal University, Niterói, Brazil; ^5^ Plastic Surgery Service, Clementino Fraga Filho University Hospital, Federal University of Rio de Janeiro, Rio de Janeiro, Brazil

**Keywords:** subcutaneous adipose tissue, superficial subcutaneous adipose tissue, deep subcutaneous adipose tissue, superficial retinacula cutis, adipose stromal/stem cells, spheroids, adipogenesis, adipokines

## Abstract

**Background:** Adipose tissue engineering may provide 3D models for the understanding of diseases such as obesity and type II diabetes. Recently, distinct adipose stem/stromal cell (ASC) subpopulations were identified from subcutaneous adipose tissue (SAT): superficial (sSAT), deep (dSAT), and the superficial retinacula cutis (sRC). This study aimed to test these subpopulations ASCs in 3D spheroid culture induced for adipogenesis under a pro-inflammatory stimulus with lipopolysaccharide (LPS).

**Methods:** The samples of abdominal human subcutaneous adipose tissue were obtained during plastic aesthetic surgery (Protocol 145/09).

**Results:** ASC spheroids showed high response to adipogenic induction in sSAT. All ASC spheroids increased their capacity to lipolysis under LPS. However, spheroids from dSAT were higher than from sSAT (*p* = 0.0045) and sRC (*p* = 0.0005). Newly formed spheroids and spheroids under LPS stimulus from sSAT showed the highest levels of fatty acid-binding protein 4 (FABP4) and CCAAT/enhancer-binding protein-α (C/EBPα) mRNA expression compared with dSAT and sRC (*p* < 0.0001). ASC spheroids from sRC showed the highest synthesis of angiogenic cytokines such as vascular endothelial growth factor (VEGF) compared with dSAT (*p* < 0.0228). Under LPS stimulus, ASC spheroids from sRC showed the highest synthesis of pro-inflammatory cytokines such as IL-6 compared with dSAT (*p* < 0.0092).

**Conclusion:** Distinct physiological properties of SAT can be recapitulated in ASC spheroids. In summary, the ASC spheroid from dSAT showed the greatest lipolytic capacity, from sSAT the greatest adipogenic induction, and sRC showed greater secretory capacity when compared to the dSAT. Together, all these capacities form a true mimicry of SAT and hold the potential to contribute for a deeper understanding of cellular and molecular mechanisms in healthy and unhealthy adipose tissue scenarios or in response to pharmacological interventions.

## Background

Two-dimensional (2D) cell culture has contributed extensively to cell biology; however, these methodologies do not replicate the complex three-dimensional (3D) *in vivo* tissue microenvironment (Fairfield et al., 2019) as observed for 3D cell culture in tissue engineering approaches (Lee et al., 2019). Adipose tissue engineering from adipose mesenchymal stem/stromal cells (ASCs) can be applied as a therapeutic autologous filling agent or as a 3D model for the understanding of diseases such as obesity and type II diabetes for drug testing. In this context, ASCs are occasionally associated with endothelial cells and macrophages in 2D and 3D cell culture (Clevenger et al., 2016; Huttala et al., 2020; Park et al., 2020; Gerlach et al., 2021).

In a 3D cell culture, the spatial arrangement of cell-to-cell and cell-to-extracellular matrix has a positive impact on crucial cell processes such as proliferation, differentiation, and various other cellular signaling events (Breslin and O’Driscoll, 2013). ASCs have an intrinsic ability to self-assemble, resulting in 3D spherical structures known as spheroids (Duguay et al., 2003; Tejavibulya et al., 2011). The self-assembly process mimics the crucial stages of embryogenesis, morphogenesis, and organogenesis (Foty et al., 1996; Dean and Morgan, 2008; Youssef et al., 2011; Bao et al., 2011). Spheroids formed from ASCs and induced to adipogenic pathways hold the potential to generate tissue models that truly mimic the human adipose tissue physiology.

Adipose tissue organoid models containing stromal vascular fraction (SVF) resident immune cell populations have contributed to immune–metabolic research (Taylor et al., 2020) and to the understanding of adipogenic differentiation (Mandl et al., 2022). After differentiation, these 3D models present a mature cellular phenotype and molecular profile similar to that of the newly isolated adipocytes (Shen et al., 2021). They also provide a more sensitive response to toxin-associated stress, compared with 2D culture, as measured by the release of cytokines and adiponectin (Klingelhutz et al., 2018). Adipose tissue SVF spheroids may also self-assemble in association with endothelial cells, producing vascularized human AT-like organoids (Muller et al., 2019). Furthermore, *in vitro* fat-on-a-chip 3D systems may be produced with critical features for functional assays of adipokine secretion, glucose uptake, and lipolysis (Bender et al., 2020).

Recently, our research group described distinct ASC subpopulations dwelling in distinct subcutaneous adipose tissue (SAT) microenvironments: superficial (sSAT), deep (dSAT), and the superficial retinacula cutis (sRC) (Baptista et al., 2021). These ASCs showed distinct differentiation commitments according to their niche of origin.

The sSAT subpopulation is described with a higher capacity for hyperplasia than the dSAT subpopulation, in particular during obesity. ASCs derived from sSAT can recapitulate this hyperplastic capacity *in vitro* (Cancello et al., 2013; Baptista et al., 2021). However, most studies on adipose tissue hyperplasia, diseases, or response to drugs employ samples from either obese (Cappellano et al., 2018) or ex-obese adipose tissue, or even from stromal vascular fractions (Boulet et al., 2013), which is a heterogeneous cell population compared with ASCs. On the other hand, ASC spheroids derived from distinct microenvironments of healthy SAT could possibly provide complex 3D *in vitro* models of SAT for the *in vitro* recapitulation of adipose tissue homeostasis according to the niche, aiming to improve the understanding of disease mechanisms and physiological responses to different drugs. Therefore, the aim of this study was to test the subpopulations of ASCs in 3D spheroid cultures induced for adipogenesis under a pro-inflammatory stimulus, assessing their capacity to recapitulate different aspects of SAT homeostasis.

## Methods

### Isolation and *in vitro* expansion of ASCs from sSAT, sRC, and dSAT

Abdominal SAT was obtained during aesthetic plastic surgeries in Brazil (Research Ethics Committee of the Clementino Fraga Filho University Hospital, Federal University of Rio de Janeiro, Protocol 145/09), as described previously (*n* = 6) (Baptista et al., 2021). The exclusion criteria considered for this study were presence of acute or chronic and/or inflammatory infectious disease, presence of type 2 diabetes mellitus, age above 18 and below 65 years, and overweight and obese patients. The superficial and deep layers were separated in the operating room immediately after adipose tissue excision. The retinacula cutis was isolated from the superficial layer in the laboratory. Each part of the adipose tissue derived from sSAT, sRC, and dSAT was fragmented into small pieces and placed in collagenase type I (Sigma) in a water bath at 37°C for 15 min and soon after centrifuged at 400 g at room temperature for 15 min. After digestion and centrifugation, the pellet was filtered in a 100-μm mesh filter. The resulting cell suspension—the SVF was seeded on the cell culture flasks and maintained at 37°C in a humid atmosphere with 5% CO_2_ to obtain the monolayers of ASCs. The ASCs were maintained in culture flasks with a chemically defined culture medium for human mesenchymal cells (MSCGM-CDTM Mesenchymal Stem Cell Medium, Chemically Defined, Lonza) supplemented with 2% fetal bovine serum (FBS), 100 μg/mL penicillin, and 100 μg/mL streptomycin (Sigma). The ASC monolayer was maintained in culture until reaching confluence, with medium changes made every 3 days. Subsequently, the monolayer was released from the culture plastic with 0.125% trypsin (Gibco) and 0.78 mM ethylenediamine tetraacetic acid (Gibco) to prepare ASC spheroids for adipogenic induction and further analysis. All ASC spheroids were formed from the ASC monolayer at the second passage. One representative donor of the adipose tissue samples was included in all analyses.

### ASC spheroid culture and adipogenic induction

ASC spheroids were produced using micro-molded non-adhesive hydrogel (2% agarose in NaCl 0.9%) with 800 μm diameter in each of the 81 circular recesses (3D Petri Dish®, MicroTissues Inc) following the manufacturer’s protocol. Approximately 2 × 10^6^ cells were seeded into the chamber of the non-adhesive hydrogel resuspended in a medium composed of DMEM supplemented with 50 ug/mL ascorbic acid (Sigma Aldrich, USA), 1.25 ug/mL human albumin (Farma Biagini SPA, Brazil), 100 ug/ml penicillin and 100 ug/ml streptomycin, and ITS 1X (Lonza). For adipogenic induction, ASC spheroids were maintained in DMEM supplemented with 50 ug/mL ascorbic acid (Sigma Aldrich, USA), 1.25 ug/mL human albumin (Farma Biagini SPA), 100 U/ml penicillin and 100 ug-ml streptomycin, and ITS 1X (Lonza, USA), and the components of adipogenic induction were 10 μM insulin (Novolin® N, Novo Nordisk, Brazil) 0.5 mM isobutylmethylxanthine, 1 μM dexamethasone, and 200 μM indomethacin (Sigma Aldrich, USA). At week 1 of adipogenic induction, the supplementation of the medium was changed, and only 10 μM insulin (Novolin® N, Novo Nordisk, Brazil) was maintained as a component of adipogenic induction for up to 5 weeks. A part of ASC spheroids induced for adipogenesis was maintained under 0.5 ug/mL lipopolysaccharide (LPS) (Sigma-Aldrich, USA).

### Diameter measurement

The diameter of the spheroids was measured from the newly formed spheroids at weeks 1 and 2 of adipogenic induction and under LPS stimulus using an optical microscope (Leica DMI 6000 B) equipped with a Leica DF 500 digital camera as described (Stuart et al., 2017). We randomly measured a total of 17 spheroids in each sample from the same micro-molded non-adhesive hydrogel. Two independent experiments were carried out for evaluation.

### Lactate dehydrogenase (LDH) release assay

LDH release was determined using the commercial non-radioactive colorimetric CytoTox 96 Cytotoxicity Kit (Promega, Madison, WI, USA). The assay was performed from the spheroids’ culture supernatant. Briefly, 30 μL of the supernatant was transferred to a 96-well plate, followed by the addition of 30 μL of the substrate. After 20 min of incubation at room temperature and in the absence of light, 30 μL of the stop solution was added to each well. The intensity of staining was proportional to the number of cells with plasma membrane disruption. The optical density (OD) was measured using a Biotek Synergy H4 microplate reader (Synergy 2, Biotek Inst., VT, USA) at 490 nm. The assay was performed using eight replicates from the supernatant of 162 spheroids obtained from two independent experiments.

### Cryosection and Nile red staining

At week 5 of adipogenic induction, ASC spheroids from sSAT, sRC, and dSAT were washed in PBS and were transferred to 4% paraformaldehyde in PBS at room temperature for 1 h. Subsequently, induced ASC spheroids were washed in PBS and placed in successive baths of 15% and 30% sucrose solution at room temperature for 24 h each. The ASC spheroids were embedded in optimal cutting temperature (OCT, Tissue-Tek) and maintained in a −80°C freezer until sectioning. Sections of 10 μm were obtained using a cryostat (Leica DMI 6000 B) and subsequently collected onto 0.01% poly-L-lysine-coated slides (Sigma). The slides with the sections were stored in a freezer at −20°C until staining. Cryosections were left at room temperature for 15 min and stained with 1 mg/mL Nile red (Sigma) diluted (1:50) in PBS. The cell nucleus was stained with 0.5 μg/mL Hoechst. The images were obtained with the aid of a fluorescence microscope (Leica DMI 6000B) (Mannheim, Germany) with LAS AF software (Leica, Mannheim, Germany). The laser microscope was programmed to stimulate at a range of 640–720 nm. Two independent analyses were evaluated, with a total of 324 spheroids of each sample obtained from four independent experiments.

### Lipolysis assay

The lipolysis assay on ASC spheroids from sSAT, sRC, and dSAT was performed using the Lipolysis Colorimetric Assay Kit (Sigma), according to the manufacturer’s instructions. Briefly, the first step consisted of preparing the standard curve. The samples (ASC spheroids) were washed with PBS and subsequently washed twice with 200 𝜇L of Lipolysis Wash Buffer. After washing, 150 𝜇L of Lipolysis Assay Buffer was added. The reaction mix was prepared and added to each well and mixed using a horizontal plate shaker and incubated for 30 min at room temperature in dark. The absorbance was measured at A570 excitation through the espectrofotômetro Synergy H4 hybrid reader. Three independent analyses in triplicate were evaluated with a total of 81 spheroids of each condition of each sample obtained from three independent experiments.

### RNA isolation, quantification, and quantitative real-time PCR (qPCR)

The expression levels of fatty acid-binding protein 4 (FABP4) and CCAAT/enhancer-binding protein-α (C/EBPα) genes of ASC spheroids from sSAT, sRC, and dSAT were measured on the newly formed spheroids, at weeks 1 and 2 of adipogenic induction and under LPS stimulus, by quantitative polymerase chain reaction (qPCR). The RNeasy Mini Kit (Qiagen, Sweden) was used for RNA extraction according to the manufacturer’s instructions. The qPCR was performed using the AgPath-ID ™ one-step RT-PCR kit (Applied Biosystems, USA). Briefly, 1.5 μl total RNA (15 ng/μl) and amplified Master Mix composed of 0.4 μL of 25x RT-PCR enzyme mix, 0.67 μl of detection enhancer, and 5 μL of 2x RT-PCR buffer and completed with RNase-free water to a final volume of 10 μL of the Master Mix solution. Subsequently, we performed the analysis of genes using specific primers and specific TaqMan probes (Applied Biosystems, USA). The analyzed genes were FABP4 (HS01086177_m1) and CEBPα (HS00269972_m1). Ribosomal protein L (RBL) (Hs99999902_m1) was set as the reference gene. For data analysis, the quantification cycle (Cq) value was determined, and specific gene expression was normalized to the reference gene using the ΔΔCq method. ASC spheroids at week 1 of adipogenic induction were relativized with the newly formed ASC spheroids (fold change week 1 of adipogenic induction *vs.* newly formed), ASC spheroids at week 1 of adipogenic induction were relativized with ASC spheroids at week 5 of adipogenic induction (fold change week 1 of adipogenic induction *vs.* week 5), and ASC spheroids of adipogenic induction were relativized with ASC spheroids of adipogenic induction under LPS at week 5 (fold change of adipogenic induction vs. LPS) to compare the difference between each pair of groups. Two independent analyses were evaluated in triplicate for each gene. RNA samples were isolated from 162 spheroids of each sample obtained from four independent experiments.

### Secretion profile of soluble mediators

Secretion analysis was performed on the culture supernatant of ASC spheroids from sSAT, sRC, and dSAT maintained in adipogenic medium at weeks 1 and 5 (with and without LPS stimulus). After 24 h of culture medium change to remove adipogenic inducers and LPS, the supernatant of all spheroid samples was harvested and frozen at −80°C. The determination of proteins was carried out using the Luminex xMAP technology based on a magnetic beads panel for recognition of human MIP-1β, IFN-y, interleukin-1ra (IL-1ra), IL-5, GM-CSF, TNFα, RANTES, IL-2, IL-1β, Eotaxin, bFGF, VEGF, PDGF-BB, IP-10, IL-13, IL-4, MCP-1, IL-8, MIP-1a, IL-10, G-CSF, IL-15, IL-7, IL-12p70, IL-17ra, and IL-9 (27-plex panel, Bio-Rad Laboratories Inc., Hercules, CA, USA) using the instrument Bio-Plex MAGPIX (Bio-Rad Laboratories Inc.). The concentration of each analyte was quantified using the software xPONENT v3.1 (LuminexCorp®, USA). The results were expressed in picograms per milliliter (ρg/ml). One independent analysis was evaluated in quadruplicate from 162 spheroids of each sample obtained from four independent experiments.

### Statistical analysis

To compare the data between sSAT, sRC, and dSAT in the analysis of diameter, the one-way ANOVA analysis test (Kruskal–Wallis test) was used followed by multiple comparisons post-test (Dunn’s test). The Omnibus normality test by D'Agostino and Pearson revealed that the lipolysis assay data are normally distributed, so the one-way ANOVA analysis test was performed followed by Sidak’s multiple comparisons test. To compare the data between sSAT, sRC, and dSAT in the PCR analysis, the two-way ANOVA analysis test was performed followed by the Tukey’s multiple comparisons test and in the multiplex analysis, the one-way ANOVA analysis test was performed followed by the Dunn’s multiple comparisons test. To compare the control group and the induced group or the group stimulated with LPS with the group not stimulated with LPS of each tissue—sSAT, sRC, and dSAT— Student’s t-test was used. The results in the graphs were realized with 95% confidence interval (95% CI: lower limit, upper limit) and expressed as mean ± standard deviation. Differences were considered statistically significant when *p* < 0.05. GraphPad Prism 6.0 software was used (GraphPad Inc., CA, USA).

## Results

### ASC spheroids from sSAT, sRC, and dSAT are responsive to adipogenesis

**FIGURE 1:**
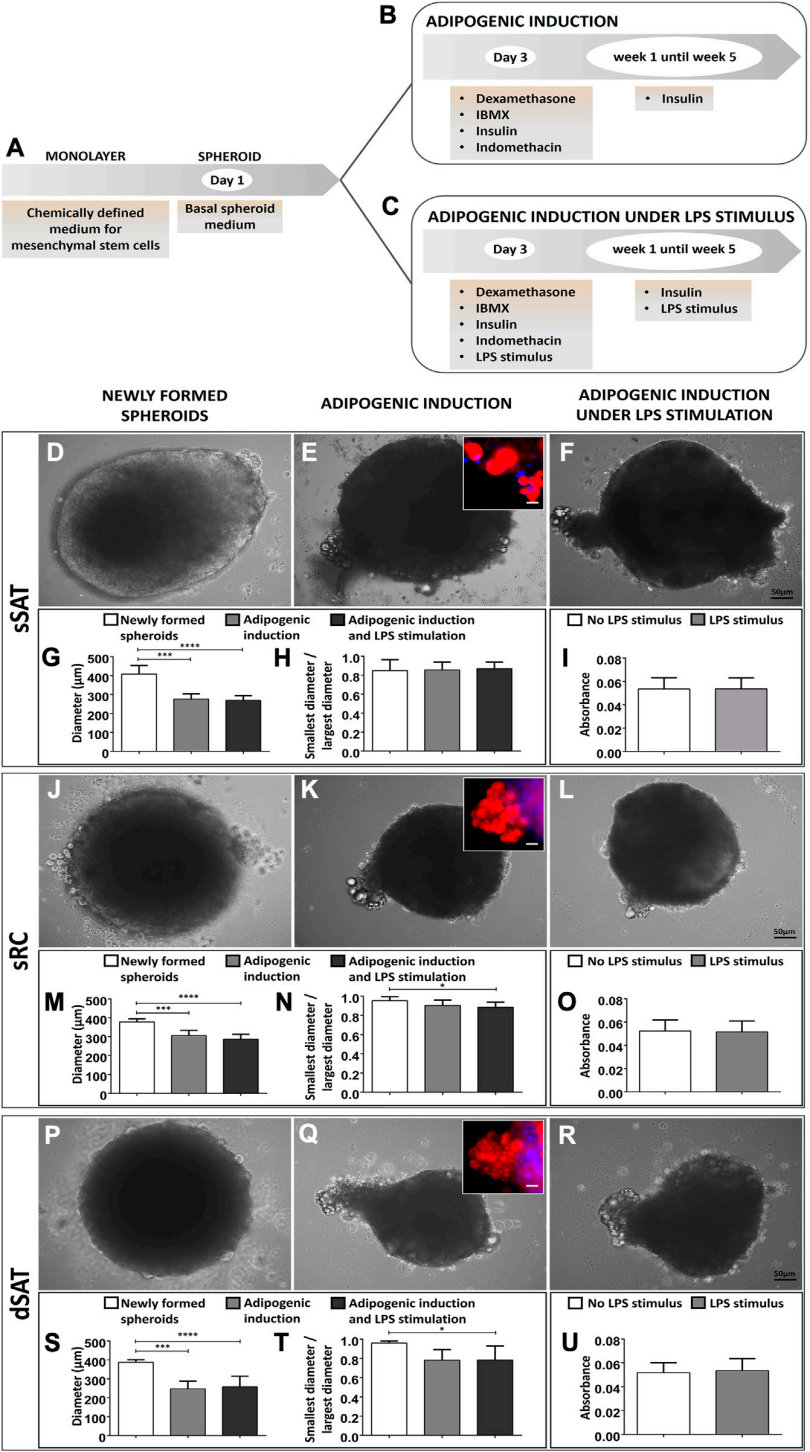
Induced ASC spheroids in the presence or absence of LPS stimulus showed lipid-accumulating cells migrating outside spheroids. Illustrative scheme of the stages of cell seeding and adipogenic induction **(A–C)**. Newly formed spheroids from sSAT presented lateralized morphology, distinct from sRC and dSAT **(D,J,P)**. Induced ASC spheroids and induced ASC spheroids under LPS stimulus at week 5 from sSAT, sRC, and dSAT showed a cell population migrating outside spheroids, resulting in a protuberance in their structure **(E,K,Q,F,L,R)**. Nile red staining revealed the presence of lipid droplets in this cell population (insets). Induced ASC spheroids in the presence or absence of LPS stimulus from sSAT, sRC, and dSAT showed similar values for this test. Two independent analyses were evaluated with a total of 324 spheroids of each sample obtained from four independent experiments. The graph measuring the diameter of the spheroids of sSAT **(G)**, sRC **(M),** and dSAT **(S)**. Graph of the ratio between the minor and major diameters of the sSAT **(H)**, sRC **(N),** and dSAT **(T)**. Induced ASC spheroids from sSAT, sRC, and dSAT showed similar levels of LDH in the absence or presence of LPS **(I,O,U)**, revealing no cytotoxic effects for this pro-inflammatory stimulus. (*p < 0.05; **p < 0.001; ***p < 0.001; ****p < 0.0001). SAT, Subcutaneous adipose tissue; ASC, Stem/stromal cells from adipose tissue. Bar size: 50 μm.

ASC spheroids were maintained in the absence of adipogenic inductors, being considered newly formed until day 3. After that, the spheroids were maintained under adipogenic inductors up to week 5 in the presence or absence of the pro-inflammatory stimulus (LPS) (Figures 1A–C). Phase contrast images revealed a lateralized morphology at day 4 in ASC spheroids from sSAT (Figure 1D) in contrast to a spheroidal morphology for sRC (Figure 1J) and dSAT (Figure 1P). All induced ASC spheroids at week 5 showed a migratory population of lipid-accumulating cells. The inset shows the Nile red staining attesting the presence of lipids in the cytoplasm of these migrating cells (Figures 1E, K, Q). Induced ASC spheroids maintained under LPS stimulus showed a similar pattern of migrating cells (Figures 1F, L, R). Induced ASC spheroids from sSAT, sRC, and dSAT reduced their initial size in approximately 100 μm at week 5 in the absence (*p* = 0.0001) or presence of LPS stimulus (*p* < 0.0001) (Figures 1G, M, S). The ASC spheroids from sSAT showed lower sphericity due to lateralization from the newly formed spheroids that remained until week 5 in both experimental conditions (in the presence or absence of LPS) (Figure 1H). The ASC spheroids from sRC (*p* = 0.0348) and dSAT (*p* = 0.0464) showed a decrease in their sphericity at week 5 of adipogenic induction exclusively under LPS stimulus compared with the newly formed spheroids (Figures 1N, T).

The induced ASC spheroids from sSAT, sRC, and dSAT showed similar levels of LDH in the absence or presence of LPS (Figures 1I, O, U), revealing no cytotoxic effects for this pro-inflammatory stimulus.

### Induced ASC spheroids under LPS stimulus from dSAT revealed the highest level of lipolysis

**FIGURE 2:**
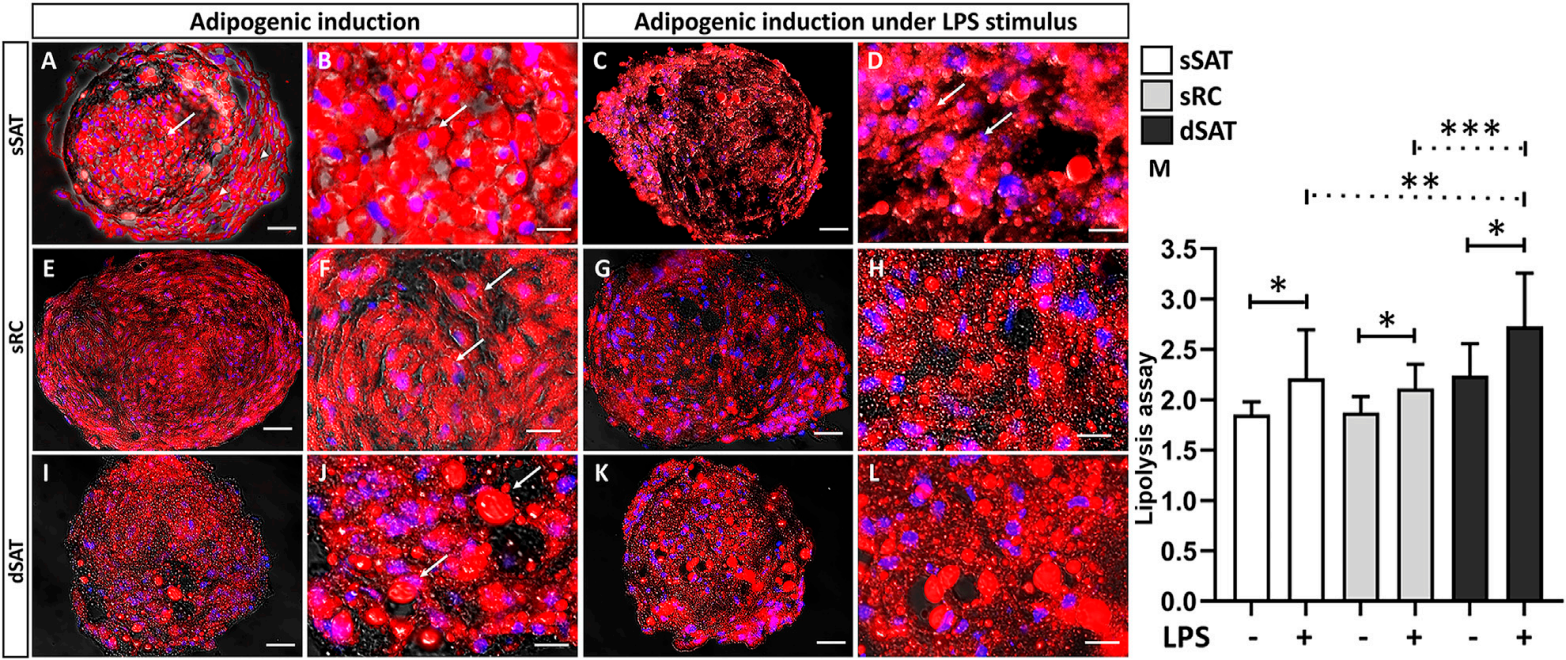
Induced ASC spheroids from sSAT showed the highest adipogenic capacity and from dSAT the highest lipolytic capacity. Induced ASC spheroids from sSAT showed a more evident presence of unilocular cells in the center of the spheroids (arrow) and more elongated cells in the periphery (arrowhead) **(A,B)**. Induced ASC spheroids from sSAT under LPS showed no difference in cell morphology **(C,D)**. Induced ASC spheroids from sRC **(E,F)** and dSAT **(I,J)** and under LPS from sRC **(G,H)** and dSAT **(K,L)** showed similar lipid accumulation. Nile red and Hoechst nuclei staining. Bar size: 50 μm. The ANOVA test evaluated the difference between the sSAT, sRC, and dSAT spheroids within each group: adipogenic induction in the absence or presence of LPS **(M)**. Three independent analyses in triplicate were evaluated with a total of 81 spheroids of each condition of each sample obtained from three independent experiments. Dashed lines indicate post-test analyses under both conditions. Solid lines indicate t-test analyses, which were performed to verify the statistical difference between induced ASC spheroids in the absence and in the presence of LPS from sSAT, sRC, and dSAT. Asterisks indicate *p*-values obtained in the post-test and in the t-test (*p < 0.05; **p < 0.001; *** *p* < 0.001). SAT, subcutaneous adipose tissue; LPS, lipopolysaccharide; ASC, stem/stromal cells from adipose tissue.

The induced ASC spheroids from sSAT showed a majority of unilocular cells with a more rounded morphology (arrow), with a preferential location in their center as well as cells located in the periphery more fibroblastic (arrowhead) (Figures 2A, B). Induced ASC spheroids from sRC (Figures 2E, F) and dSAT (Figures 2I, J) showed multilocular cells, evident by the large amount of small lipid droplets and rounded nuclei (arrow). Under LPS stimulus, all ASC spheroids maintained their response to adipogenic inductors (Figures 2C, D, G, H, K, L). Interestingly, the induced ASC spheroids from sSAT showed a more evident presence of multilocular cells (arrow) (Figure 2D) compared with spheroids maintained in the absence of LPS (Figure 2B).

We evaluated the lipolytic capacity of induced ASC spheroids as a functional assay. Induced ASC spheroids under LPS stimulus from sSAT (*p* = 0.0268), sRC (*p* = 0.0157), and dSAT (*p* = 0.0116) showed an increase in their lipolytic capacity compared with spheroids maintained in the absence of LPS. Interestingly, the induced ASC spheroids from dSAT under LPS stimulus showed the highest lipolytic capacity compared with sSAT (*p* = 0.0045) and sRC (*p* = 0.0005) (Figure 2M).

### ASC spheroids from sSAT showed the highest levels of FABP4 and CEBPα mRNA expression under LPS stimulus during adipogenic induction

**FIGURE 3:**
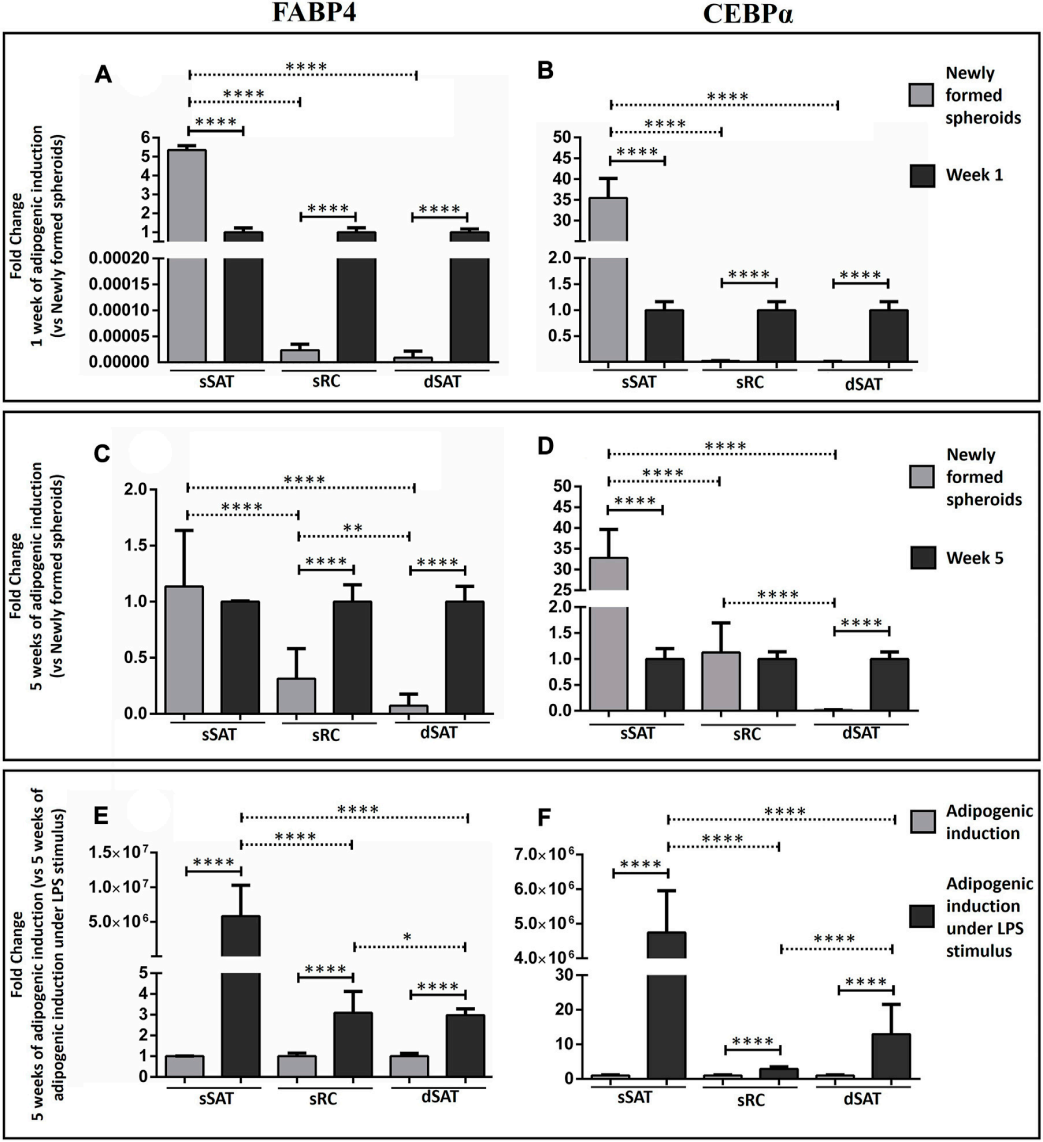
Analysis of FABP4 and CEBPα revealed that the ASC spheroids from sSAT showed higher expression of both genes from the newly formed spheroids and induced spheroids for the adipogenic pathway under LPS stimulus when compared to sRC and dSAT. Graph of the relativization of the newly formed spheroids in relation to spheroids with 1 week of adipogenic induction (Fold change 1 week of adipogenic induction *vs.* newly formed spheroids) of FABP4 gene expression **(A)** and CEBPα gene expression **(B)**. Graph of the relativization of the newly formed spheroids in relation to spheroids with 5 weeks of adipogenic induction (Fold change 5 week of adipogenic induction *vs.* newly formed spheroids) of FABP4 gene expression **(C)** and CEBPα gene expression **(D)**. Graph of the relativization of spheroids induced by the adipogenic pathway in relation to the spheroids induced by the adipogenic pathway under LPS stimulation at week 5 (Fold change 5 weeks of adipogenic induction *vs.* 5 weeks of adipogenic induction under LPS stimulus) of FABP4 gene expression **(E).** CEBPα gene expression **(F)**. Two independent analyses were evaluated in triplicate for each gene. RNA samples were isolated from 162 spheroids of each sample obtained from two independent experiments. The two-way ANOVA test followed by the Tukey multiple comparison analysis evaluated the difference between the evaluated conditions and between the sSAT, sRC, and dSAT. (*p < 0.05; **p < 0.001; ***p < 0.001; **** *p* < 0.0001). SAT, subcutaneous adipose tissue; LPS, lipopolysaccharide; ASC, stem/stromal cells from adipose tissue; FABP4, fatty acid-binding protein 4; C/EBPα, CCAAT/enhancer-binding protein-α.

As expected, adipogenic inductors increased *FABP4* and *CEBPα* gene expression in ASC spheroids from sRC and dSAT compared with newly formed spheroids from the respective samples (*p* < 0.0001) (Figures 3A, B). Curiously, the newly formed ASC spheroids from sSAT showed a higher FABP4 and CEBPα gene expression than induced ASC spheroids from the respective sample (*p* < 0.0001) (Figures 3A, B).

LPS stimulus was capable of increasing FABP4 and CEBPα gene expression in all ASC spheroids compared with adipogenic induction (*p* < 0.0001). Interestingly, only ASC spheroids from sSAT showed an increase up to 5 million-fold in their gene expression (Figures 3E, F).

### Induced ASC spheroids from sRC showed greater synthesis of angiogenic cytokines and MCP-1 compared to the dSAT

**FIGURE 4:**
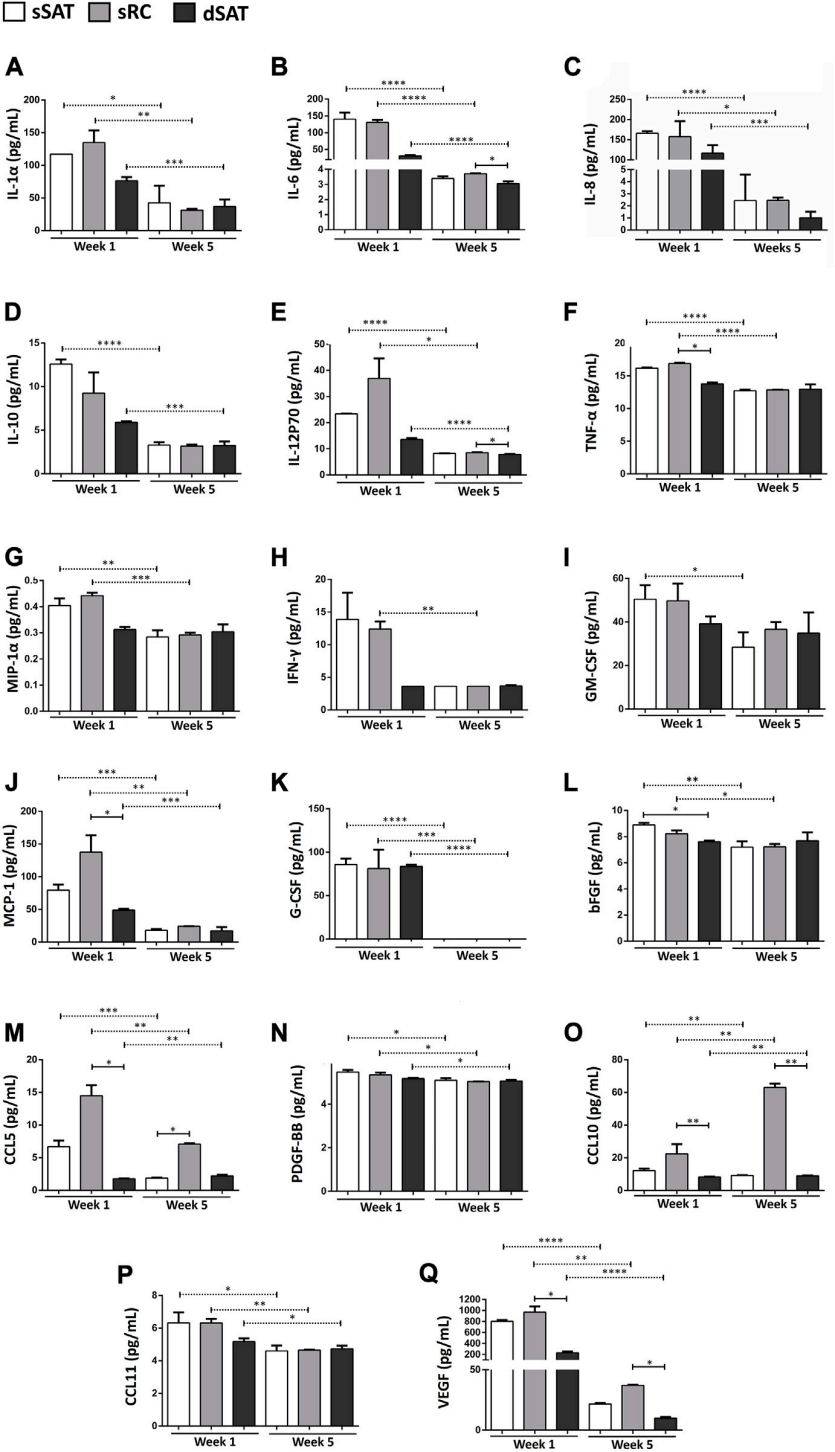
ASC spheroids from sRC showed higher secretion of CCL5, CCL10, and VEGF after week 5 of adipogenic induction compared to sSAT and dSAT. Secretion of IL-1α **(A)**, IL-6 **(B)**, IL-8 **(C)**, IL-10 **(D)**, IL-12p70 **(E)**, TNF-α **(F)**, MIP-1α **(G)**, IFN-γ **(H)**, GM-CSF **(I)**, MCP-1 **(J)** G-CSF **(K)**, bFGF **(L)**, CCL5 **(M)**, PDGF-BB **(N)**, CCL10 **(O),** CCL11 **(P)**, and E VEGF **(Q)** of ASC spheroids in weeks 1 and 5 of adipogenic induction from sSAT, sRC, and dSAT. One independent analysis was evaluated in quadruplicate from 162 spheroids of each sample obtained from four independent experiments. Data are expressed as mean ± SD. The ANOVA test evaluated the difference between ASC spheroids from sSAT, sRC, and dSAT within each group: weeks 1 and 5 of adipogenic induction. Dashed lines indicate post-test analyses under both conditions. Solid lines indicate t-test analyses, which were performed to verify the statistical difference between week 1 of adipogenic induction and week 5 of adipogenic induction from sSAT, sRC, and dSAT. Asterisks indicate *p* values obtained in the post-test and in the t-test (* *p* < 0.05; **p < 0.001; ***p < 0.001; **** *p* < 0.0001). SAT, subcutaneous adipose tissue; ASC, stem/stromal cells from adipose tissue; IL, interleukin; IL-6, interleukin-6; IL-8, interleukin-8; IL-10, interleukin-10; IL-12p70, interleukin-12; IL-15, interleukin-15; IFN-γ, interferon-γ; MCP-1, monocyte chemoattractant protein-1; bFGF, basic fibroblast growth factor; VEGF, vascular endothelial growth factor; GM-CSF, granulocyte-macrophage colony-stimulating factor; G-CSF, granulocyte colony-stimulating factor; PDGF-BB, platelet-derived growth factor; and CCL, CC chemokine ligand.

The induced ASC spheroids from sSAT, sRC, and dSAT significantly decreased the secretion at week 5 of adipogenic induction of the following cytokines: IL-1Rα (Figure 4A), IL-6 (Figure 4B), IL-8 (Figure 4C), IL-12p70 (Figure 4E), MCP-1 (Figure 4J), G-CSF (Figure 4K), CCL5 (Figure 4M), PDGF-BBM (Figure 4N), CCL10 (Figure 4O), CCL11 (Figure 4P), and VEGF (Figure 4Q). Induced ASC spheroids from sRC showed the highest secretion capacity for MCP-1 and CCL5 compared with spheroids at week 1 from dSAT (*p* < 0.024 and *p* < 0.0257, respectively). At week 5, the highest secretion was detected for CCL10 compared with spheroids from dSAT (*p* < 0.0257). At weeks 1 and 5 of adipogenic induction, VEGF secretion in ASC spheroids from sRC was higher than that from dSAT (*p* < 0.0257 and *p* < 0.0228, respectively). No difference was found in the secretion of IL-2, IL-5, and IL-7 among the three types of ASC spheroids, as well as no difference in secretion at weeks 1 and 5 of induction (data not shown).

### Induced ASC spheroids from sRC showed greater synthesis of angiogenic cytokines and of pro-inflammatory cytokines under LPS stimulus compared to the dSAT

**FIGURE 5:**
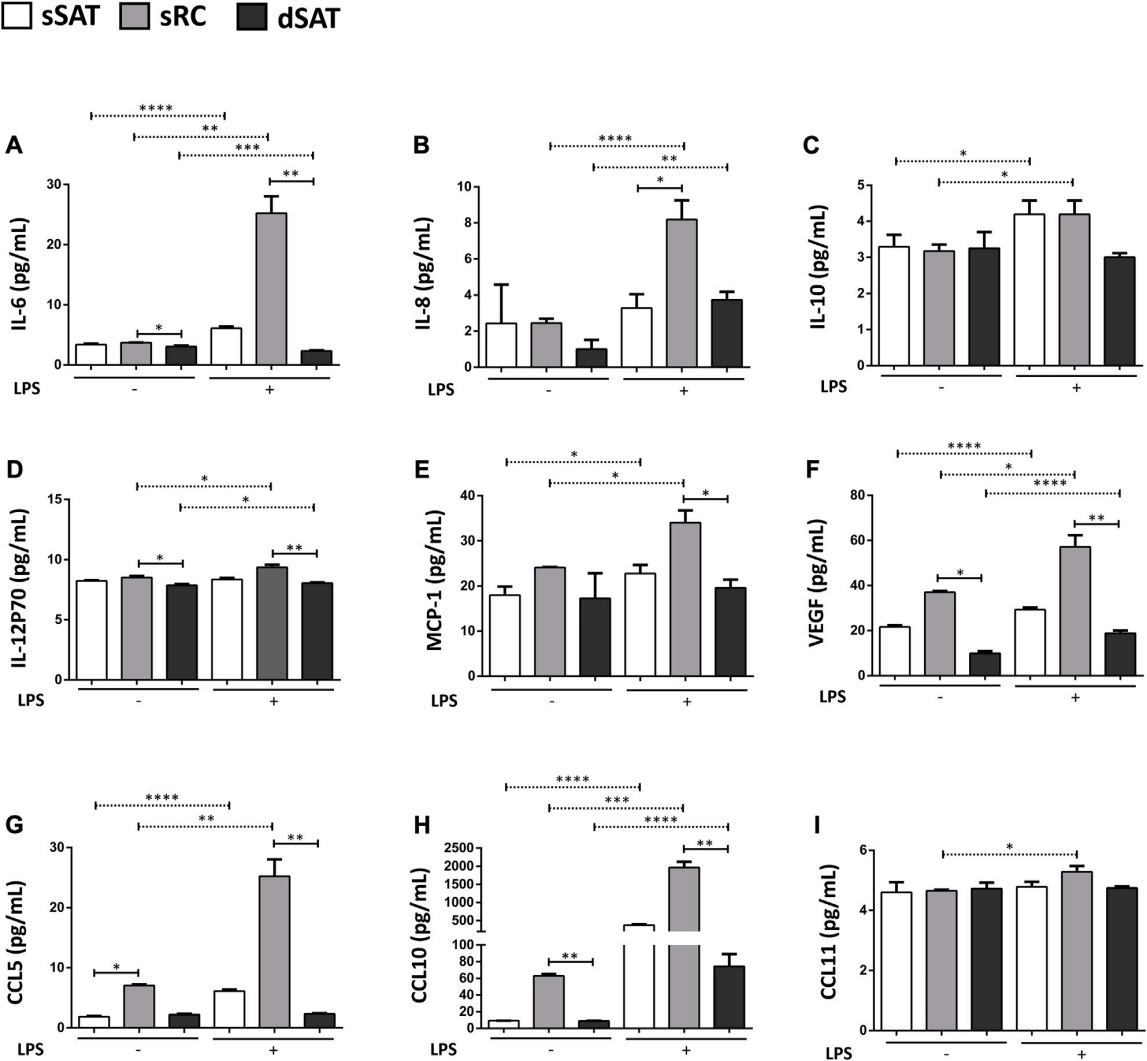
ASC spheroids from sRC were more responsive to LPS stimulus compared with sSAT and dSAT. Secretion of IL-6 **(A)**, IL-8 **(B)**, IL-10 **(C)**, IL12p70 **(D)**, MCP-1 **(E)**, VEGF **(F)** CCL5 **(G)**, CCL10 **(H)**, and CCL11 **(I)** of induced spheroids for the adipogenic pathway under LPS stimulus from sSAT, sRC, and dSAT. One independent analysis was evaluated in quadruplicate from 162 spheroids of each sample obtained from four independent experiments. Data are expressed as mean ± SD. The ANOVA test evaluated the difference between ASC spheroids from sSATl, sRC, and dSAT within each group: induced spheroids for the adipogenic pathway under LPS stimulus. Dashed lines indicate post-test analyses under both conditions. Solid lines indicate t-test analyses, which were performed to verify the statistical difference between induced spheroids for the adipogenic pathway under LPS stimulus from sSAT, sRC, and dSAT. Asterisks indicate p values obtained in the post-test and in the t-test (*p < 0.05; **p < 0.001; ***p < 0.001; **** *p* < 0.0001). SAT, subcutaneous adipose tissue; LPS, lipopolysaccharide; ASC, stem/stromal cells from adipose tissue; IL, interleukin; IL-6, interleukin-6; IL-8, interleukin-8; IL-10, interleukin-10; IL-12p70, interleukin-12; IL-15, interleukin-15; IFN-γ, interferon-γ; MCP-1, monocyte chemoattractant protein-1; bFGF, basic fibroblast growth factor; VEGF, vascular endothelial growth factor; GM-CSF, granulocyte-macrophage colonyQ18 stimulating factor; G-CSF, granulocyte colony stimulating factor; PDGF-BB, platelet-derived growth factor; CCL, CC chemokine ligand.

Induced ASC spheroids from sRC showed higher secretion compared with sRC in the absence of LPS stimulus of IL-6 (Figure 5A) *p* < 0.001, IL-8 (Figure 5B) *p* < 0.0001, IL-10 (Figure 5C) *p* < 0.0431, IL-2P70 (Figure 5D) *p* < 0.0166, MCP-1 (Figure 5E) *p* < 0.0158, VEGF (Figure 5F) *p* < 0.001, CCL5 (Figure 5G) *p* < 0.05, CCL10 (Figure 5H) *p* < 0.001, and CCL11 (Figure 5I) *p* < 0.05. Interestingly, the induced ASC spheroids under LPS stimulus from sRC showed higher secretion compared with dSAT of the IL-6 (Figure 5A) *p* < 0.0092, IL-12p70 (Figure 5D) *p* < 0.0092, MCP-1 (Figure 5E) *p* < 0.0172, VEGF (Figure 5F) *p* < 0.0228, CCL5 (Figure 5G) *p* < 0.0092, and CCL10 (Figure 5H) *p* < 0.0092. The induced ASC spheroids from sSAT, sRC, and dSAT showed no difference in the secretion of IL-1b, IL-1Rα, IL-2, IL-7, IFN-γ, GM-CSF, GCS-F, PDGF-BB, and bFGF, even when exposed to the pro-inflammatory stimulus of LPS (data not shown).

## Discussion

Recently, we showed, for the first time in scientific literature, the presence of ASCs in a poorly explored human SAT Q11 microenvironment, the superficial retinacula cutis (sRC) (Baptista et al., 2021). In this study, adipogenic-induced ASC spheroids from dSAT showed the greatest lipolytic activity, while those from sSAT presented the greatest adipogenicity and those from sRC presented the greatest secretory capacities. According to our results, distinct physiological properties of SAT can be recapitulated in ASC spheroids, serving as a complex 3D model for the understanding of healthy and unhealthy adipose tissue homeostasis.

Under stimulus, the ASC spheroids undergo proliferation and differentiation processes, triggering migration and selection events that can grow in a different structure, which leads to a symmetry break. In this way, these spheroids can acquire a certain degree of architectural complexity to mimic the organization of organs *in vivo* (Hofer and Lutolf, 2021). Induced ASC spheroids from sSAT showed a prominent cellular concentration in their core with a lighter periphery along with a lateralized morphology at day 4, as in other models, such as intestinal organoids (Sato et al., 2009), minibrains (Sutcliffe and Lancaster, 2017), or chondrogenic ASC spheroids (Côrtes et al., 2021). On the other hand, the induced ASC spheroids from sRC and dSAT maintained a spheroidal morphology. Lancaster and colleagues also report budding on the surface of brain cell constructs, with cells migrating out of the main brain organoid mass (Sutcliffe and Lancaster, 2017). Similarly, at week 5 of adipogenic induction, all spheroids started to show projections of lipid droplets on their surface, even in the presence of LPS stimulus.

The scientific literature describes visceral adipose tissue as being more lipolytic than SAT (Garaulet et al., 2006). The SAT acts primarily as an organ of stock of triglycerides due to the mechanism of hyperplasia (Verboven et al., 2018), acting as metabolic buffer for energy excess (McQuaid et al., 2011). Similar to the entire SAT, the sSAT is described as a protective fat deposit in patients with type 2 diabetes (Golan et al., 2012), while visceral and dSAT share similarities. Monzon and collaborators reported dSAT as a more lipolytic active tissue compared with sSAT (Monzon et al., 2002). The tissue lipotoxicity observed in visceral adipose tissue and dSAT occurs due to the hypertrophy of adipocytes (Verboven et al., 2018), whereas impaired adipogenesis of visceral and dSAT can make the storage of energy excess difficult, resulting in triglyceride overflow (McQuaid et al., 2011). Our results support this hypothesis, revealing that the ASC spheroids from dSAT are more lipolytic than sRC and sSAT. Interestingly, the ASC spheroids from all microenvironments of SAT analyzed in this study increased their lipolytic capacity under pro-inflammatory (LPS) stimulus. The increase of lipolytic capacities of visceral and SAT during obesity were widely described in scientific literature (Ibrahim, 2010).

During adipogenic differentiation, the master regulator genes PPARγ and CEPBα are expressed in the stage in which cells accumulate lipids in their cytoplasm as unilocular or multilocular droplets. Both the genes induce transcription of many adipocyte genes and play a role in maintaining the adipocyte phenotype (Ntambi and Kim, 2000). Surprisingly, the newly formed ASC spheroids from sSAT showed increased expression of CEPBα and FABP4, a marker of adipocyte progenitors (Soukas et al., 2001) compared with induced ASC spheroids from sSAT at weeks 1 and 5. This result revealed a spontaneous commitment to adipogenic differentiation in the ASC spheroids from sSAT. As expected, the ASC spheroids from sRC and dSAT showed higher expression of CEPBα only at week 1 of adipogenic induction compared with the newly formed spheroids. In a previous study, we have reported that ASC monolayers from sSAT also show a higher expression of CEBPα and FABP4 compared with sRC and dSAT. In addition, other authors also described a hyperplastic profile from sSAT of obese individuals (Cancello et al., 2013) and from preadipocytes of healthy individuals (Walker et al., 2008).

In this study, the ASC spheroids were stimulated with LPS to mimic an inflammatory condition observed in some diseases such as obesity. Interestingly, we observed that all ASC spheroids showed higher expression of FABP4 and CEPBα when exposed to LPS stimulus. Furthermore, induced ASC spheroids from sSAT maintained their higher expression of CEPBα and FABP4 compared with sRC and dSAT. The systemic inflammatory scenario inhibits adipogenic differentiation in obese patients (Isakson et al., 2009), mainly through the inhibition of PPARγ (Keophiphath et al., 2009; Gregor and Hotamisligil, 2011). However, the different adipose tissue depots have their intrinsic capacities. One of our previous studies showed that ASCs from visceral adipose tissue have a greater capacity to secrete pro-inflammatory cytokines together with a lower adipogenic potential. On the other hand, ASCs from SAT have a greater adipogenic potential and lower secretion of pro-inflammatory cytokines (Silva et al., 2015). Furthermore, during the initial stage of obesity, hyperplasia of adipose tissue allows a greater ability to assimilate fatty acids, therefore representing a “healthier” growth without the exacerbated increase in the secretion of pro-inflammatory cytokines synthesized by hypertrophic adipocytes (Chan and Hsieh, 2017). Our hypothesis is that when the sSAT is exposed to an inflammatory condition, its hyperplastic capacity contributes to the protection of the entire adipose tissue, in an attempt to mitigate the inflammatory condition. Further studies will confirm if this condition was recapitulated in ASC spheroids.

The cytokine IL-6 is a pleiotropic inflammatory cytokine (Nishimoto and Kishimoto, 2006), for which several studies have reported an involvement in angiogenesis under physiological as well as pathological conditions (Hashizume et al., 2009; Tzeng et al., 2013). This involvement has been reported mainly by the fact that IL-6 promotes VEGF expression (Jee et al., 2004; Gertz et al., 2012; Huang et al., 2016). In this study, induced ASC spheroids from sRC showed the highest levels for IL-6 and VEGF compared to dSAT. Di Taranto and collaborators previously reported a higher expression of VEGF in sSAT compared to the dSAT, however, without including the sRC niche (Di Taranto et al., 2015). In the present study, we dissected the sRC from sSAT, generating ASC spheroids from these distinct microenvironments, subsidizing the hypothesis that the sRC supports the angiogenesis from sSAT.

In addition, the inflammatory stimulus provided by LPS triggered an increase in VEGF, CCL10, and CCL11 secretion of ASC spheroids from sRC. TNF-α increases the expression of the chemokine CCL5 and its receptor CCR5 in adipose tissue (Tourniaire et al., 2013) and in the central nervous system (Bachelerie et al., 2014). In fact, at the beginning of adipogenic differentiation, we had a greater secretion of TNF-α from ASC spheroids from sRC when compared to dSAT, supporting their role in angiogenesis. Furthermore, under the LPS stimulus, we observed an increase in secretion of CCL5 in ASC spheroids from sRC. CC chemokines are related to angiogenesis in inflammation-induced pathologies. These chemokines are known to indirectly promote angiogenesis from the recruitment of macrophages to the site of inflammation, which, in turn, will secrete factors that trigger the formation of new vessels. Supporting this hypothesis, ASC spheroids from sRC showed the highest secretion of MCP-1 compared to dSAT at the onset of adipogenic differentiation and after LPS-induced inflammation.

Interestingly, besides MCP-1, the induced ASC spheroids from sRC showed higher secretion of IL-6 and IL-12p70 under LPS stimulus compared with dSAT. Stromal cells, including ASCs, when exposed to an inflammatory scenario, have the ability to polarize, moving from an anti-inflammatory to a pro-inflammatory phenotype (Silva et al.; Baptista et al., 2015; Le Blanc and Davies, 2015). Recently, dSAT has been correlated with obesity-associated complications in a similar way to visceral adipose tissue. Kim and collaborators showed a positive correlation between serum levels of inflammatory cytokines and adipokines in dSAT (Kim et al., 2016). In samples from obese patients, inflammatory genes were overexpressed in the dSAT (Cancello et al., 2013), different from the present study, where ASCs from dSAT were isolated from healthy donors. In addition, it is important to emphasize the critical role of basal inflammation in tissue regeneration and repair (Cooke, 2019). Our hypothesis is that the secretion of pro-inflammatory cytokines of ASC spheroids from sRC serves to remodel the adipose tissue since these microenvironments had greater adipogenic and lower lipolytic capacities compared with dSAT. Additional *in vivo* studies are needed to attest this hypothesis.

The main limitation of the study was that all experiments were carried out with one representative donor adipose tissue sample. The pilot experiments were performed with samples from more than one donor; however, it was observed that the samples presented the same profile of biological responses. This homogeneity of samples is probably due to the exclusion criteria used in this study.

## Conclusion

ASCs from sSAT, sRC, and dSAT were able to self-assemble to generate spheroids in a scalable model of the 3D cell culture. Compared to the dSAT, ASC spheroids from sSAT and sRC are in favor of stimulating adipogenesis and angiogenesis, respectively. While the inflammatory stimulus triggers lipolysis in all ASC spheroids, dSAT is the most lipolytic subpopulation. Together, all these capacities result in a true mimicry of SAT and hold the potential to contribute for a deeper understanding of cellular and molecular mechanisms in healthy and unhealthy scenarios in response to drug stimulus. This 3D spheroid model also opens opportunities for evaluating individual responses in personalized medicine. Last, but not the least, the present study contributes and emphasizes the importance of considering the superficial and deep subcutaneous adipose tissue as two distinct adipose tissue depots and also revealed the potential relevance of the retinacula cutis, a cell niche of SAT not yet well-studied in the scientific literature.

## Data Availability

The datasets presented in this study can be found in online repositories. The names of the repository/repositories and accession number(s) can be found in the article/Supplementary Material.

## Abbreviations

SAT, subcutaneous adipose tissue; sSAT, superficial SAT; dSAT, Deep SAT; sRC, superficial retinacula cutis; SVF, stromal-vascular fraction; ASCs, adipose-derived stromal/stem cells; PBS, phosphate-buffered saline; MSCGM-CDTMs, chemically defined culture medium for human mesenchymal cells; qPCR, quantitative polymerase chain reaction; VEGF, vascular endothelial growth factor; FABP4, fatty acid-binding protein 4; C/EBPα, CCAAT/enhancer-binding protein-α; IL, interleukin; IL-6, interleukin-6; IL-8, interleukin-8; IL-10, interleukin-10; IL-12p70, interleukin-12; IL-15, interleukin-15; IFN-γ, interferon-γ; MCP-1, monocyte chemoattractant protein-1; bFGF, basic fibroblast growth factor; GM-CSF, granulocyte–macrophage colony-stimulating factor; G-CSF, granulocyte colony-stimulating factor; PDGF-BB, platelet-derived growth factor; CCL, CC chemokine ligand; LPS, lipopolysaccharide; CAPES, Coordination for the Improvement of Higher Education Personnel; FAPERJ, Carlos Chagas Filho Foundation for Research Support of the State of Rio de Janeiro.
